# Scale for the assessment and rating of ataxia (SARA): Übersetzung und kulturelle Anpassung an den deutschsprachigen Raum

**DOI:** 10.1007/s10354-023-01014-8

**Published:** 2023-04-24

**Authors:** Julia Silberbauer, Sonja Schidl, Gudrun Diermayr, Tanja Schmitz-Hübsch, Andrea Greisberger

**Affiliations:** 1 Klinische Abteilung für Neurologie, Universitätsklinikum Tulln, Tulln, Österreich; 2 Karl Landsteiner Privatuniversität für Gesundheitswissenschaften, Krems, Österreich; 3 Fachhochschule Burgenland GmbH, Eisenstadt, Österreich; 4 SRH Hochschule Heidelberg, Fakultät für Therapiewissenschaften, Heidelberg, Deutschland; 5 Experimental and Clinical Research Center, Max Delbrück Center for Molecular Medicine in the Helmholtz Association and Charité – Universitätsmedizin Berlin, Berlin, Deutschland; 6 NeuroCure Clinical Research Center, Charité – Universitätsmedizin Berlin, Freie Universität Berlin und Humboldt-Universität zu Berlin, Berlin, Deutschland; 7 Department Gesundheitswissenschaften, FH Campus Wien, Wien, Österreich

**Keywords:** Neurologie, Ataxie, Assessment, Übersetzung, Kulturelle Anpassung, Neurology, Ataxia, Assessment, Translation, Cultural adaptation

## Abstract

**Hintergrund/Ziel:**

Die Scale for the Assessment and Rating of Ataxia (SARA) ist ein praxistaugliches Assessment für die Einschätzung des Schweregrades einer Ataxie und für die Evaluierung von Therapiemaßnahmen. Um im deutschsprachigen Raum über eine entsprechend internationalen Vorgaben übersetzte Version zu verfügen, war das Ziel dieser Arbeit, die SARA ins Deutsche zu übersetzen und für den deutschsprachigen Raum kulturell anzupassen.

**Methode:**

Der Übersetzungsprozess beinhaltete 6 Schritte. Dabei wurde die Verständlichkeit der Übersetzung in Interviews mit den späteren Nutzer*innen überprüft.

**Ergebnisse:**

Neun Physiotherapeut*innen und sechs Ärzt*innen mit unterschiedlichen Arbeitsumgebungen wurden interviewt. Sieben Personen waren in Deutschland und je vier in Österreich bzw. der Schweiz tätig. Die Interviews führten zu einer Präzisierung der übersetzten Version. Im länderspezifischen Vergleich wurden keine Auffälligkeiten der Verständlichkeit festgestellt.

**Schlussfolgerung:**

Mit dieser Arbeit ist eine von der Mitautorin der Originalpublikation autorisierte deutsche Version der SARA verfügbar. Die Ergebnisse liefern außerdem methodische Erkenntnisse zum Übersetzungsprozess von beobachtungsbasierten standardisierten Assessments.

**Zusatzmaterial online:**

Zusätzliche Informationen sind in der Online-Version dieses Artikels (10.1007/s10354-023-01014-8) enthalten.

## Abstract (en)

**Background/objective:**

The scale for the assessment and rating of ataxia (SARA) is a feasible assessment for the classification and evaluation of therapeutic interventions. In order to provide access to the SARA in German, the aim of this work was to translate the SARA into German and to adapt it according to international guidelines for German-speaking countries.

**Method:**

The process involved six steps. The comprehensibility of the scale was assessed using interviews with potential users.

**Results:**

A total of nine physiotherapists and six physicians working in various clinical settings were interviewed, seven of them worked in Germany and four each in Austria and Switzerland. The interviews led to a refined version of the translation. The comprehensibility testing revealed no country-specific differences.

**Conclusion:**

A German version of the SARA authorized by the co-author of the original publication, is now available. The results provide methodological insights into the translation process of observation-based standardized assessments.

## Einleitung

Der Begriff Ataxie wird in der aktuellen neurologischen Terminologie meist zur Beschreibung degenerativer Erkrankungen und Läsionen des Kleinhirns und der zerebrozerebellären Verbindungsbahnen verwendet [1, 2]. Ataxie beeinflusst willkürliche Bewegungen, Haltungskontrolle, Sprechen, Schlucken und/oder auch die Augenbewegungen und kann in Form einer Stand- oder Gangataxie, Rumpf- oder Extremitätenataxie auftreten [3, 4].

In der Literatur werden unterschiedliche Klassifikationen von Ataxien beschrieben. Es lassen sich vereinfacht 3 ätiologische Gruppen unterscheiden: (1) hereditäre Formen wie die Friedreich-Ataxie oder die spinozerebelläre Ataxie (SCA), (2) sporadische (idiopathische) Formen und (3) symptomatische Formen infolge von umschriebenen Läsionen bei Blutung, Ischämie, Tumor oder Abszess im Zentralnervensystem [1]. Aktuell gibt es nur wenige Untersuchungen zur Prävalenz von Ataxie. Laut der „Leitlinie für Diagnostik und Therapie in der Neurologie“ der Deutschen Gesellschaft für Neurologie (DGN) zeigen Ataxien eine Prävalenz von etwa 10–20:100.000 [5]. Zu den symptomatischen und sporadischen Formen liegen bisher keine Daten vor. Die Häufigkeit von symptomatischen Ataxien wird lediglich in Zusammenhang mit multipler Sklerose (MS) beschrieben: Bis zu 27,6 % der Personen mit MS geben Ataxiesymptome an [6]. Neben der pharmakologischen Behandlung der Ataxie werden Physiotherapie, Ergotherapie und Logopädie als die 3 Hauptsäulen der Behandlung der Ataxie genannt [5, 7, 8]. Assessments zur Beurteilung von Ataxie konzentrieren sich auf die motorischen Funktionen [9]. Da das Symptom Ataxie vielfältig und häufig in Kombination mit weiteren neurologischen Symptomen auftritt, sind die Diagnostik und die Evaluation der Behandlung besonders herausfordernd.

Im Sinne einer evidenzbasierten Praxis tragen standardisierte, auf ihre psychometrischen Eigenschaften überprüfte, klinische Assessments zur differenzierten Analyse eines Gesundheitsproblems und in weiterer Folge zu einer zielgerichteten, patient*innenorientierten Behandlung bei [10]. Mit standardisierten Assessments können Einschränkungen der Körperfunktionen und -strukturen, der Aktivitäten oder der Partizipation identifiziert und quantifiziert werden. Des Weiteren werden sie zur Evaluation, Diagnose- und Prognosestellung verwendet [11]. Wesentlich für die standardisierte Durchführung eines Assessments sind kurze und prägnante Erläuterungen der Durchführung und der Bewertung der Untersuchungsergebnisse. Ihre regelmäßige Verwendung wird gefördert durch einen leichten Zugang zu Assessments [12, 13]. Mangelnde Englischkenntnisse wurden im internationalen Raum dagegen als Barriere für die Implementierung von evidenzbasierter Praxis identifiziert [14–16]. Daher besteht die Forderung, standardisierte Assessments nach internationalen Richtlinien in die Erstsprache der jeweiligen Benutzer*innen zu übersetzen und anschließend auf ihre psychometrischen Eigenschaften hin zu untersuchen [17, 18]. Dies gilt auch für klinische Assessments [19–21].

Bezogen auf die Ataxie, stehen verschiedene standardisierte Assessments zur Auswahl: Die am häufigsten genutzten sind „International Cooperative Ataxia Rating Scale“ (ICARS), die „Scale for the Assessment and Rating of Ataxia“ (SARA) oder auch die „Friedreich’s Ataxia Rating Scale“ (FARS) [22–24]. Die ICARS wurde als semiquantitative Skala zur Erfassung des klinischen Schweregrades der zerebellären Ataxie eingeführt, erwies sich jedoch aufgrund des erhöhten Zeitaufwandes von mehr als 30 min als zu zeitintensiv für den klinischen Alltag [1, 22, 23, 25]. Ebenso wurde die ICARS für einen inkonsistenten Skalenaufbau mit ungleich gewichteten Testuntereinheiten kritisiert [26]. Während die ICARS für Testung der Ataxie im Allgemeinen einsetzbar ist, ist die FARS ausschließlich für die Bewertung der Friedreich-Ataxie konzipiert [27] und soll nicht bei Personen mit Ataxie anderer Ursachen angewendet werden. Die SARA wurde mit dem Anliegen entwickelt, eine praxistaugliche Skala für alle Formen der Ataxie zu gestalten. Sie ist ein semiquantitatives, ordinalskaliertes Assessment mit 8 Items, wobei die einzelnen Items eine unterschiedliche Anzahl von Bewertungsstufen aufweisen (9 Bewertungsstufen für Item 1; 7 Bewertungsstufen für Items 2 und 4 sowie 5 Bewertungsstufen für Items 3 und 5–8). In der Bewertung können maximal 40 Punkte erreicht werden. Bei der Beurteilung der Ataxie der oberen und unteren Extremität (Items 5–8) wird seitengetrennt bewertet und anschließend der Mittelwert berechnet, der in den Summenscore eingeht. Die Beurteilung der einzelnen Items wird abschließend summiert, wobei 0 Punkte bedeutet, dass keine Ataxie vorhanden ist, und 40 Punkte für eine schwere Form von Ataxie stehen [22].

Die SARA ist für ein breites Spektrum von leicht bis schwer betroffenen Personen mit Ataxie geeignet und kann in unterschiedlichen Phasen des Krankheitsverlaufs angewendet werden. Sie zeichnet sich durch eine hohe interne Konsistenz und Linearität sowie eine hohe Intra- und Intertester-Reliabilität bei Personen mit SCA aus [22, 28]. Des Weiteren zeigen Untersuchungen zur Kriteriumsvalidität eine starke Korrelation der SARA zur ICARS und FARS [22, 29, 30]. Die SARA kann außerdem zwischen Personen, die eine Ataxie aufweisen, und gesunden Kontrollpersonen unterscheiden [22, 31]. Dass die SARA nicht nur für Personen mit hereditären Formen der Ataxie ein reliables und valides Messinstrument ist, sondern auch bei symptomatischen Formen der Ataxie eingesetzt werden kann, wurde beispielsweise von Kim et al. (2011), Salci et al. (2017) und Weyer et al. (2007) untersucht und bestätigt [20, 32, 33]. Der SARA Gesamtscore ändert sich signifikant im Laufe der Erkrankung [22, 33]. Yamauchi et al. (2021) belegten die prädiktive Validität und die Responsivität der Skala bei Personen mit Ataxie nach einem akuten Schlaganfall [34]. Referenzwerte für die Interpretation von Veränderung liegen für Personen mit SCA und Friedreich-Ataxie vor [35, 36]. Die SARA kann – mit minimalen Limitationen – auch bei Kindern und Jugendlichen mit Ataxie angewendet werden [37, 38].

Aufgrund der zahlreichen Untersuchungen zur SARA ist diese für den Praxisalltag ein vielversprechendes Assessment für die Evaluation aller 3 Formen der Ataxie. Aktuell gibt es keine nach internationalen Richtlinien [18, 39] übersetzte deutsche Version der SARA. Um die Verwendung der SARA im deutschsprachigen Raum zu fördern, war das Ziel der vorliegenden Arbeit, die SARA vom Englischen ins Deutsche zu übersetzen und für den deutschsprachigen Kontext interkulturell anzupassen. Außerdem wurde erhoben, ob berufsgruppen- oder länderspezifische Unterschiede bezüglich der Verständlichkeit vorliegen.

## Material und Methoden

Der Übersetzungsprozess wurde von der Mitautorin der Originalpublikation Tanja Schmitz-Hübsch (TSH) begleitet und orientierte sich an den Empfehlungen von Beaton et al. (2000) ([18]; Tab. 1). Auf Anfrage bei der zuständigen Ethikkommission der burgenländischen Krankenanstalten (burgenländische Krankenanstalten Gesellschaft m.b.H Bereich Qualitätsmanagement Burgenland in Eisenstadt) meldete diese zurück, dass für die vorliegende Studie kein Votum erforderlich sei.

**Table 1:** 

Schritt 1	Juli 2019	Vorwärtsübersetzung durch 3 unabhängige Übersetzer*innen ins Deutsche
Schritt 2	August 2019	Zusammenführung der Vorwärtsübersetzungen
Schritt 3	September 2019	Rückübersetzung der Zusammenführung durch 3 unabhängige Übersetzer*innen ins Englische Erstellung der *Synthese*
Schritt 4	Oktober 2019	Expert*innenkomitee mit Einigung auf eine *Arbeitsversion*
Schritt 5	Januar bis April 2020	Verständlichkeitsprüfung mit Ärzt*innen und Physiotherapeut*innen Erstellung der *finalen Version* unter Absprache mit der Mitautorin der Oritinalpublikation
Schritt 6	Mai 2020	Abschließende Durchsicht der *deutschen Version der SARA* durch die Mitautorin der Originalskala

*SARA* „Scale for the assessment and rating of ataxia“

### Übersetzungsprozess

In Schritt 1 wurde die Originalversion von einer Englischlehrerin einer Sekundarstufe (Übersetzerin ohne medizinisches oder therapeutisches Vorwissen), einer Physiotherapeutin und einer Ergotherapeutin (Übersetzerinnen mit medizinischem und klinischem Hintergrund) unabhängig voneinander vom Englischen ins Deutsche übersetzt. In Schritt 2 wurden die 3 Übersetzungen von 2 Autorinnen (Andrea Greisberger AG, Julia Silberbauer JS) zusammengeführt. Anschließend fand die Rückübersetzung der Zusammenführung (Schritt 3) durch 2 Rückübersetzer mit Englisch als Erstsprache und einem Englischlehrer einer Sekundarstufe statt. Alle 3 Rückübersetzer waren betreffend die Originalversion der SARA verblindet und arbeiteten unabhängig voneinander. Die Rückübersetzungen wurden von 2 Autorinnen (AG, JS) dem Original gegenübergestellt, Abweichungen wurden besprochen, und eine Synthese wurde erstellt. Im anschließenden Expert*innenkomitee (Schritt 4) wurden alle Versionen der Übersetzungen sowie die Gleichwertigkeit der Übersetzung zum Original überprüft und aufgetretene Diskrepanzen besprochen. Das Komitee bestand aus einem Methodiker, der Mitautorin der Originalpublikation (TSH), den 3 Vorwärtsübersetzerinnen, 1 Rückübersetzer und 2 Physiotherapeutinnen (eine Physiotherapeutin mit Lehr- und Forschungserfahrung, eine Physiotherapeutin mit Erfahrung in der Behandlung von Personen mit Ataxie). Das Expert*innenkomitee wurde von der Erstautorin (JS) geleitet. Die im Konsens erstellte Arbeitsversion wurde anschließend (Schritt 5) einer Verständlichkeitsprüfung unterzogen.

### Verständlichkeitsprüfung

In Schritt 5 des Übersetzungsprozesses wurden leitfadengestützte Interviews mit Ärzt*innen und Physiotherapeut*innen durchgeführt.

Die Interviewpartner*innen wurden von den Autorinnen mittels gezielter Kontaktaufnahme in der Schweiz, Deutschland und Österreich rekrutiert. Eingeschlossen wurden (1) Ärzt*innen und Physiotherapeut*innen aus dem Fachbereich Neurologie mit den Berufsabschlüssen Diplom/Staatsexamen, Bachelor, Master und/oder Doktorat/PhD, die (2) in den Arbeitssettings Akutkrankenhaus, stationäre Rehabilitationseinrichtung, ambulante Tagesklinik oder in einer freien Praxis arbeiten. Ebenso mussten sie (3) in Deutschland, Österreich oder der Schweiz tätig sein, (4) Deutsch als Erstsprache sprechen und (5) innerhalb von 2 Jahren mindestens 5 Personen mit Ataxie untersucht oder behandelt haben. Ausgeschlossen wurden (1) Personen mit < 2 Jahren Berufserfahrung und (2) Personen, die bereits eine nicht autorisierte deutsche Version der SARA verwenden. Die Auswahl der Interviewpartner*innen erfolgte mittels bewussten Stichprobenplans nach unterschiedlichen soziodemografischen Kriterien: (1) Land, in dem die Personen tätig sind, (2) Jahre der Berufserfahrung, (3) Ausbildungsgrad, (4) Berufsgruppe, (5) Arbeitssetting und (6) Kenntnis der englischen Originalversion der SARA. Ziel war es, bezüglich dieser Kriterien eine möglichst hohe Diversität zu erlangen. Die Interviewpartner*innen erhielten vorab ein ausführliches Informationsblatt zur Studie und wurden gebeten, eine Einverständniserklärung zu unterzeichnen.

Die Interviews wurden im Jänner 2020 per Telefon (JS) anhand eines Interviewleitfadens durchgeführt. In den Interviews wurden Methoden wie die Technik des Lauten Denkens, Nachfragetechniken betreffend der Verständlichkeit und die Paraphrasierungstechnik nach Lenzner et al. (2015) angewendet [40]. Den Interviewpartner*innen wurde 2 Werktage vor dem Interview die Arbeitsversion der SARA mit dem Hinweis, diese vor dem Interview nicht zu lesen, per E‑Mail zugeschickt. Der Interviewleitfaden wurde nicht versandt, da keine Vorbereitung auf das Interview nötig bzw. erwünscht war. Nach Einverständnis der Interviewpartner*innen wurden Audioaufzeichnungen der Interviews gestartet und zusätzlich handschriftliche Notizen während des Interviews erstellt.

Die aufgenommenen Audiodateien und die handschriftlich erstellten Notizen wurden von 2 Personen (AG, JS) unabhängig voneinander mittels vorab strukturierten Auswertungsschemas in ein selektives Protokoll transkribiert. Nach Empfehlung von Mayring (2016) diente dieses selektive Protokoll der Reduktion von großen Materialmengen auf das Wesentliche [41]. Bezüglich der Verständlichkeit wurden folgende Punkte protokolliert: (1) ob Textteile auf Anhieb verstanden wurden (ja/nein/teilweise/nicht beurteilbar), (2) ob die Verständlichkeit aufgrund der Paraphrasierung und Worterklärung deutlich wurde (ja/nein/teilweise/nicht beurteilbar), (3) warum/was nicht verstanden wurde und (4) wurden genannte „Verbesserungsvorschläge“ notiert.

Die ersten beiden Punkte zur Verständlichkeit wurden anschließend zusammenfassend quantitativ ausgewertet. Bei Diskrepanzen wurde auf Basis der beiden Protokolle und der Audiodateien ein Konsens im Rahmen einer Diskussion zwischen JS und AG gefunden. Um die Verständlichkeit der Items untereinander vergleichbar zu machen, wurde die Verständlichkeit prozentual berechnet [42]: Jedes von insgesamt 8 Items besteht aus einer Anleitung und der Beschreibung der einzelnen Bewertungsstufen. Die Anzahl der „Ja“-Einschätzungen der Anleitungen und der Bewertungsstufen jedes Items wurde addiert und durch die Anzahl der Bewertungsstufen inklusive der Anleitung dividiert. So wurde die Verständlichkeit pro Item (Anleitung und Bewertungsstufen) für alle Interviewpartner*innen gesamt, aber auch in Bezug auf Berufsgruppen und Nationalitäten berechnet.

Mittels adaptierter qualitativer Inhaltsanalyse in Form der inhaltlichen Strukturierung nach Mayring (2015) wurden Punkt 3 (Probleme der Verständlichkeit) und Punkt 4 (Verbesserungsvorschläge) des oben beschriebenen Protokolls analysiert und dargestellt [43]. Das anfänglich deduktive Vorgehen wurde durch ein induktives Vorgehen ergänzt [43–45]. Anhand der Forschungsfragen wurden zu Beginn die Hauptkategorien „Verständlichkeit“ und „Verbesserungsvorschläge“ (inklusive Unterkategorien) definiert. Beim ersten Materialdurchlauf (unabhängig durchgeführt von JS und AG) kristallisierte sich für beide Autorinnen heraus, dass eine zusätzliche Hauptkategorie „Praktikabilität“ hilfreich ist. Alle Interviews wurden von JS und AG mithilfe dieser vorläufigen Haupt- und Unterkategorien unabhängig voneinander analysiert und anschließend im Konsens zusammengeführt. Diese Zusammenführung wurde unter regelmäßiger Miteinbeziehung des Interviewmaterials im Autorinnenteam diskutiert und weiter präzisiert [44, 45].

Anhand der Auswertung der Interviews wurden Änderungen an der Übersetzung der Skala vorgenommen. Die Verständlichkeit dieser Version wurde in einer Feedbackschleife mit den Interviewpartner*innen und der Mitautorin der Originalpublikation der SARA per E‑Mail evaluiert. Im Anschluss wurde die übersetzte und kulturell angepasste deutsche Version der Mitautorin der Originalstudie (TSH) zur abschließenden Durchsicht vorgelegt (Schritt 6).

## Ergebnisse

### Übersetzungsprozess

Unsicherheiten, welche bei der Übersetzung und Zusammenführung der Vorwärts- und Rückübersetzungen auftraten, wurden im Expert*innenkomitee besprochen. Dies betraf v. a. die Übersetzung des Titels „Speech“, die einheitliche Verwendung von „eindeutig“, „Wanken“, „Schwanken“, „dauerhaft“, „durch einen Arm“, „natürliche Position“, „bewertet“ und „Finger-Folge Versuch“. Das Expert*innenkomitee identifizierte bestimmte Ausdrücke, deren Verständlichkeit in den Interviews überprüft werden sollte (z. B. „Tandemgang“, „durch einen Arm“). Die geschlechtsneutrale Übersetzung von „Proband“, in „Person“, die im Syntheseprozess nach der Vorwärtsübersetzung gewählt wurde, wurde vom Expert*innenkomitee für passend befunden und findet sich auch in der deutschen Version der Skala wieder.

Die vom Expert*innenkomitee erarbeitete Arbeitsversion wurde der Mitautorin der Originalpublikation (TSH) vorgelegt. Diese empfahl, weitere Formulierungen in den Interviews zu überprüfen (z. B. „permanent“ oder „konstant“ oder „anhaltend“, „Kontakt bleibt erhalten“). Die Details zu den Veränderungen durch das Expert*innenkomitee und durch die Mitautorin der Originalpublikation (TSH) in diesem Schritt können im Anhang (Supplemental File 1) nachgelesen werden.

### Verständlichkeitsprüfung

Fünfzehn Interviews wurden im Januar 2020 via Telefon durchgeführt. Die Dauer der Interviews betrug im Durchschnitt 50 min (±14,2), wobei das kürzeste Interview 31 min und das längste 92 min in Anspruch nahm. Das Durchschnittsalter der Interviewpartner*innen betrug 40,1 Jahre (±10,5), und sie hatten durchschnittlich 15,9 Jahre (±9,5) Berufserfahrung. Sieben Interviewpartner*innen kamen aus Deutschland, je 4 aus Österreich und der Schweiz, wobei 3  Interviewpartner*innen nicht in der Schweiz aufgewachsen sind, aber seit 18 bis 28 Jahren in der Schweiz leben. Weitere Details können in Tab. 2 nachgelesen werden.

**Table 2:** 

		DE	AUT	CH	Gesamt
Anzahl Interviewpartner*innen [*n*]	–	7	4	4	15
Geschlecht [*n*]	Weiblich	6	3	1	10
	Männlich	1	1	3	5
Alter [*n*]	< 30 Jahre	2	1	–	3
	30 bis 45 Jahre	4	1	1	6
	> 45 Jahre	1	2	3	6
Berufserfahrung [*n*]	< 5 Jahre	–	1	–	1
	5 bis 20 Jahre	6	2	1	9
	> 20 Jahre	1	1	3	5
Berufsgruppe [*n*]	Ärzt*in	4	1	1	6
	Physiotherapeut*in	3	3	3	9
Ausbildungsgrad [*n*]	Doktor/PhD	3	2	1	6
	Habilitation	1	–	–	1
	Diplom/Staatsexamen	1	1	–	2
	Bachelor	2	1	1	4
	Master	–	–	2	2
Arbeitssetting [*n*] ^a^	Akutkrankenhaus	4	1	1	6
	Stationäre Rehab-Einrichtung	2	1	2	5
	Ambulante Tagesklinik	1	1	–	2
	Freie Praxis	–	–	1	1
	Forschung und Lehre	1	1	–	2
Vorwissen [*n*]	Skala unbekannt	2	2	2	6
	Englische Version bekannt, wird aber nicht verwendet	2	2	2	6
	Englische Version bekannt und wird verwendet	3	–	–	3

*n* Anzahl der Interviewpartner*innen, *DE* Deutschland, *AUT* Österreich, *CH* Schweiz

^a^Mehrfachantworten möglich

Die Interviews wurden bezüglich der Verständlichkeit der einzelnen Items (Anleitung inklusive Bewertungsstufen) quantitativ ausgewertet. Die Überschrift der Skala wurde von allen Interviewpartner*innen verstanden. Ebenfalls waren einzelne Bewertungsstufen bei einzelnen Items für alle verständlich: Bewertungsstufe 2, 3, 4 und 8 von Item 1, Bewertungsstufe 2 von Item 2, Bewertungsstufe 0 von Item 3, Bewertungsstufen 0, 2, 6 von Item 4, Bewertungsstufen 0, 2, 3 und 4 von Item 7. Dem gegenüber steht, dass keine Anleitung von allen Interviewpartner*innen verstanden wurde. Zusammengefasst wurde das Item 7 mit 97,8 % am besten und das Item 5 mit 85,6 % am schlechtesten verstanden. Die länder- und berufsgruppenspezifische Auswertung der Verständlichkeit ist in Tab. 3 dargestellt.

**Table 3:** 

	Gesamt	DE	AUT	CH	Ärzt*innen	PT
Item 1: Gang (9 Bewertungsstufen)	90,7	91,4	95,0	85,0	91,7	90,0
Item 2: Stand (7 Bewertungsstufen)	86,7	83,9	90,6	87,5	91,7	83,3
Item 3: Sitzen (5 Bewertungsstufen)	91,1	90,5	95,8	87,5	86,1	94,4
Item 4: Sprechstörung (7 Bewertungsstufen)	95,8	96,4	90,6	100,0	97,9	94,4
Item 5: Finger-Folge-Versuch (5 Bewertungsstufen)	85,6	81,0	83,3	95,8	80,6	88,9
Item 6: Finger-Nase-Versuch (5 Bewertungsstufen)	95,6	92,9	95,8	100,0	91,7	98,1
Item 7: Schnelle wechselnde Handbewegungen (5 Bewertungsstufen)	97,8	97,6	100,0	95,8	97,2	98,1
Item 8: Knie-Hacke-Versuch (5 Bewertungsstufen)	93,3	88,1	100,0	95,8	100,0	88,9

0 bedeutet, Anleitung und keine Bewertungsstufe dieses Items wurde von irgendeiner interviewten Person verstanden, 100 bedeutet, Anleitung und alle Bewertungsstufen wurden von allen interviewten Personen verstanden

*AUT* Österreich, *CH* Schweiz, *DE* Deutschland, *PT* Physiotherapeut*innen

Die abschließend definierten 5 Hauptkategorien (Probleme der Verständlichkeit, Probleme in der Anwendung, Verbesserungsvorschläge, Layout, Sprache) und deren Unterkategorien sind in Tab. 4 dargestellt.

**Table 4:** 

**Probleme der Verständlichkeit**	
Zu viel Inhalt	Anleitung zu lange (Item 1, 2, 5, 6 und 7) Mehr als eine Aufgabe und/oder eine Messgröße wird in einem Item überprüft (Item 1, 2, 7) Zu viele Bewertungsstufen (Item 1, 2 und 4) Bewertung unklar, da mehr als eine Messgröße beurteilt wird (Item 1, 2, 3 und 7)
Ungenaue Formulierung	Definition von Schlüsselbegriffen unklar (z. B. natürliche Position, normale Haltung, bequem sitzen) Anleitung kann erst verstanden werden, wenn die Bewertungsstufen gelesen werden (Item 1, 2, 5 und 6) Fehlende Informationen z. B. zu Hilfsmittelverwendung in der Bewertung Diskrepanz zwischen Anleitung und Bewertungsstufen (Item 5 und 6)
**Probleme in der Anwendung**	
Berufsalltag	Berufsalltag nicht mit den Anforderungen der Skala vereinbar (z. B. barfuß stehen nicht erlaubt) Ähnliche Tests in der ärztlichen Untersuchungsroutine, dadurch Abänderung der Anleitung und Bewertungsstufen (z. B. Item 6 und 7) Ärztliche Untersuchungsräume teilweise zu klein für 10 m Gehstrecke PTs hinterfragen verstärkt Ausdrücke wie „Unterstützung durch einen Arm“, „natürliche Position“, „normales Sitzen“, „posturale Kontrolle“ oder „Propriozeption“ Testen der Sprache und des Sprechens ungewöhnlich für PTs PTs assoziieren den Begriff „Tremor“ nicht mit Ataxie – dadurch entsteht Verwirrung
Theoretisches und praktisches Vorwissen	Erfahrung aus der praktischen Tätigkeit mit Personen mit Ataxie wirft unterschiedliche Fragen auf Erfahrung aus praktischer Tätigkeit stellt Anleitungen und Bewertungsstufen infrage Wissen zur klassischen Testtheorie Wissen zu Haltung und Bewegung Wissen zu sensomotorischer Kontrolle PTs fragten, ob die SARA posturale Kontrolle, Gleichgewichtsreaktionen oder Propriozeption valide testet
**Verbesserungsvorschläge**	
Betreffend die Übersetzung	Ergänzende Wörter, z. B. Skala für *das* Assessment …, *modifizierter* Finger-Nase-Versuch, über *alle* 3 Wiederholungen Kürzung durch Verwendung von Abkürzungen Umstellung des Satzbaus, z. B. bei Item 1 und 2 Veränderung der Wortwahl, z. B. Ausweichschritt statt Fehlschritt Streichen von Wörtern, z. B. Titel von Item 4 Sprechen/Sprechstörung Formatierung, z. B. Formatierung der Mittelwertberechnung
Betreffend das Original	Nur eine Aufgabe testen in Item 1 und 2 Klarheit über Schlüsselbegriffe Abgleich zwischen Anleitung und Bewertungsstufen Integration von Testungen ohne visuelle Kontrolle Anzahl der Bewertungsstufen für alle Items gleich; eventuell Dichotomisierung (Person kann es, kann es nicht) Bewertung von nur einer Messgröße Ergänzendes Manual anfertigen, z. B. zur genaueren Beschreibung der Items und der Bewertungsstufen, Art der Anleitung (verbal und/oder vorzeigen), Notwendigkeit einer Einschulung Praxisfreundliche Beschreibungen, z. B. 10 Zyklen in 10 s leichter einschätzbar als 10 Zyklen in 7 s Überdenken der Mittelwertberechnung, v. a. bei Personen mit Hemiataxie Skala testet hauptsächlich die ICF-Komponenten Körperfunktionen und -strukturen, die Komponenten Aktivitäten und Partizipation wären alltagsrelevanter
**Layout**	Ähnlichkeit des Layouts mit englischem Original positiv Kompaktes Format auf 2 Seiten positiv Mittelwertberechnung muss deutlicher hervorgehoben werden Anleitung und Bewertungsstufen im Formular praktisch
**Sprache**	Verständlichkeit im Allgemeinen gut, nur „Rosinenpickerei“ Verwendung von gegenderten Ausdrücken wird positiv hervorgehoben

*ICF* „International Classification of Functioning Disability and Health“

#### Hauptkategorie Probleme der Verständlichkeit

In dieser Kategorie wurden Probleme der Verständlichkeit in Zusammenhang mit *zu viel Inhalt* in der Anleitung und/oder in den Bewertungsstufen geäußert (z. B. InterviewpartnerIn 1 zu Item 5 und 7 „die Anleitungen sollten gekürzt werden, weniger Text wäre besser“). Zu einem schlechten Verständnis trugen auch *ungenaue Formulierungen* von Schlüsselbegriffen wie „natürliche Position“ und „normale Haltung“ bei (z. B. Interviewpartner*in 14 zu Item 2: „Was bedeutet natürliche Position? Ist das ein hüftbreiter oder spontaner Stand? Ist es schulterbreit oder eine normale Spurbreite? So etwas wie eine natürliche Position gibt es in der Neuro nicht!“).

#### Hauptkategorie Probleme in der Anwendung

Unter Probleme in der Anwendung der Skala wurden Aussagen kategorisiert, die entweder aufgrund des *Berufsalltags* oder aufgrund von *theoretischem und praktischem Vorwissen* zu Herausforderungen in der Anwendung der Skala führen könnten.

Probleme in der Anwendung aufgrund des *Berufsalltags* wurden v. a. in Zusammenhang mit eingespielten Alltagsroutinen und Abläufen genannt. Das Item 8 ist das einzige Item, das im Liegen auszuführen ist, und wurde hinterfragt (Interviewpartner*in 6 „Wäre eine Testung im Sitz auch erlaubt?“). Die Bezeichnung „schnelle, alternierende Handbewegungen“ in Item 7 führte zu einer Verwechselung mit der Dysdiadochokinesetestung. Ebenso wurde die vom standardisierten Finger-Nase-Versuch abweichende Durchführung des Items 6 nicht immer erkannt. Für die Beurteilung des Gangs in Item 1 sollte eine 10-m-Gehstrecke zur Verfügung stehen, wofür v. a. ärztliche Untersuchungsräume häufig zu klein sind.

Erfahrungen und Vorwissen aus der praktischen Tätigkeit mit Personen mit Ataxie, die Fragen bezüglich der Anwendung aufwarfen, wurden in der Unterkategorie *theoretisches und praktisches Vorwissen* zusammengefasst. So wurde z. B. die Frage gestellt, wie man damit umgehen soll, wenn aufgrund einer erhöhten Spastik Barfuß-Stehen (Item 2) nicht möglich ist. Interviewpartner*in 3 fragte: „Welcher Fuß muss beim Tandemstand vorne stehen, wenn eine Hemiataxie vorliegt?“, oder Interviewpartner*in 14 meinte: „Verfälscht die Mittelwertberechnung denn nicht den Gesamtscore bei Hemiataxiepatient*innen?“ Ebenso wurde angemerkt, dass sich die Items auf Körperstrukturen und -funktionen der International Classification of Functioning Disability and Health (ICF) fokussieren und die ICF-Komponenten Aktivitäten und Partizipation fehlen.

#### Hauptkategorie Verbesserungsvorschläge

Die gesammelten Verbesserungsvorschläge bezogen sich einerseits auf die *Übersetzung*, andererseits betrafen diese auch das *Original*. Durch die Verbesserungsvorschläge, die die *Übersetzung* betrafen, konnten in der finalen Version der Skala Konkretisierungen im Text vorgenommen werden: Wörter wurden gestrichen (z. B. im Titel von Item 4), und die Wortwahl wurde verändert (z. B. Ausweichschritt statt Fehltritt).

Die Verbesserungsvorschläge, die in die Übersetzung nicht aufgenommen werden konnten, weil sie eine Veränderung des Originals bedeutet hätten, wurden in der zweiten Unterkategorie (*Betreffend Original*) gesammelt. Zu Item 1 und 2 wurde vorgeschlagen, dass das Testen einer Aufgabe ausreichend ist (Interviewpartner*in 1 zu Item 1 und 2 „Ich würde hier nur eine Aufgabe testen.“). Ebenfalls bei Item 1 wurde ein Abgleich zwischen der Anleitung und den Bewertungsstufen bezüglich der 10-m-Gehstrecke und der 10 Schritte angeregt. Die unterschiedliche Anzahl an Bewertungsstufen wurde ebenfalls angemerkt (Interviewpartner*in 6 „Fürs statistische Weiterarbeiten wäre es besser, die gleiche Anzahl an Bewertungspunkten bei jedem Item zu verwenden, denn jedes Item sollte gleich relevant sein!“). Eine praxisfreundliche Beschreibung wurde betreffend Item 5 und 6 angeregt (Interviewpartner*in 14 „Die cm-Angaben sollten gestrichen werden, diese sind mit freiem Auge nicht messbar, und es ist nicht alltagstauglich, diese mit einem Lineal zu messen.“). Auch Item 7 könnte in der Praxis leichter beurteilt werden, wenn die Bewertungsstufen anders formuliert wären (Interviewpartner*in 6 „Es wäre besser, nur die Dysmetrie in leicht-mittel-schwer einzuteilen.“).

#### Hauptkategorie Layout

In der Kategorie Layout wurde positiv angemerkt, dass die Form der Übersetzung dem englischen Original sehr ähnlich ist.

#### Hauptkategorie Sprache

Die Übersetzung wurde als allgemein gut verständlich beschrieben, und die angesprochenen Problematiken wurden als „Rosinenpickerei“ (Interviewpartner*in 10) kategorisiert. Die Verwendung von geschlechtsneutralen Ausdrücken wurde begrüßt.

Die Details zu den sprachlichen Veränderungen im Laufe des Prozesses können im Anhang 1 (Supplemental File 1) nachgelesen werden.

### Finalisierung der deutschen Übersetzung

Die finale Version der Skala wurde an die interviewten Personen und an die Mitautorin der Originalpublikation der SARA mit der Bitte um Rückmeldung versendet. Aufgrund der Rückmeldung der Mitautorin der Originalpublikation der Skala (TSH) wurden noch folgende Änderungen vorgenommen: In der Anleitung der Items 1, 2 und 3 wurde „durchgehend“ mit „konstant“ ersetzt, und in der Anleitung des Item 7 wurde „Abfolge“ mit „Wiederholung“ ausgetauscht. Ebenfalls in Item 7 wurde ergänzt, dass es sich um die Pro- und Supination der Hand handelt. Sechs von den 15 Interviewpartner*innen meldeten zurück, dass die adaptierte Skala nun besser verständlich sei als die Version, die für die Interviews vorlag. Neun Interviewpartner*innen antworteten nicht auf die Anfrage.

Die finale und von der Mitautorin der Originalpublikation (TSH) freigegebene deutsche Version der SARA ist in Anhang 2 (Supplemental File 2) zu finden.

## Diskussion

Die SARA wurde in der vorliegenden Studie nach internationalen Richtlinien übersetzt. Nach dem Übersetzungsprozess wurden 15 Personen für die Verständlichkeitsprüfung rekrutiert. Die Ergebnisse der Interviews führten zu sprachlichen Anpassungen der Übersetzung. Die Interviews waren nicht nur zielführend für eine sprachliche Optimierung der Übersetzung. Sie machten auch allgemeine Anwendungsschwierigkeiten (z. B. barfuß stehen und gehen, 10-m-Gehstrecke, Interpretation von Schlüsselbegriffen) deutlich und gaben so Hinweise auf die Inhaltsvalidität der Skala. Länderspezifische Verständlichkeitsprobleme konnten nicht identifiziert werden; berufsgruppenspezifische Unterschiede wurden in der Interpretation der qualitativen Daten beobachtet.

### Übersetzungsprozess

Bei der Durchführung des Übersetzungsprozesses der SARA wurden international empfohlene Richtlinien berücksichtigt [18, 39, 46, 47]. Beaton et al. (2000) empfehlen mindestens 2 Übersetzer*innen pro Stufe, in der vorliegenden Studie wurden 3 Vorwärtsübersetzer*innen und 3 Rückwärtsübersetzer*innen eingesetzt [18]. Drei Übersetzer*innen stellten sich in den Phasen der Zusammenführung der Vorwärtsübersetzungen bzw. bei der Überprüfung der Rückübersetzungen als Bereicherung dar, da sprachliche Diskrepanzen leichter ausgeräumt werden konnten.

Bezüglich der Rückübersetzungen empfehlen Beaton et al. (2000) und Guillemin et al. (1993) Übersetzer*innen ohne medizinischen Hintergrund [18, 46]. Begründet wird dies von Guillemin et al. (1993) damit, dass Rückübersetzer*innen unvoreingenommen und ohne Erwartungshaltung übersetzen sollten [46]. Unsere Erfahrung zeigt allerdings, dass Rückübersetzer*innen ohne medizinischen Hintergrund Begriffe, die für medizinisch geschulte Personen eindeutig verständlich sind, anders bzw. fachfremd interpretieren (z. B. Ataxie mit „dizziness“ oder Gang mit „hallway or movement“). Die Empfehlungen von Guillemin et al. (1993) beziehen sich auf die Übersetzung von Instrumenten zur Messung der gesundheitsbezogenen Lebensqualität, die primär von Patient*innen selbst ausgefüllt werden und keine medizinischen Fachbegriffe beinhalten [46]. Bei der Übersetzung eines klinischen Assessments, das medizinische Fachbegriffe beinhaltet, weisen unsere Erfahrungen darauf hin, dass Rückübersetzer*innen ohne medizinischen Hintergrund Schwierigkeiten mit medizinischem Vokabular haben. Daher könnte der gezielte Einsatz von Rückübersetzer*innen mit medizinischem Hintergrund im Fall der Übersetzung eines klinischen Assessments zielführender sein.

### Verständlichkeitsprüfung

Im länderspezifischen Vergleich konnten keine Besonderheiten festgestellt werden. Im bewussten Stichprobenplan wurden die Interviewpartner*innen unter anderem nach dem Land, in dem die Personen tätig sind, ausgewählt. Hierbei muss kritisch angemerkt werden, dass 3 von 4 Interviewpartner*innen, die in der Schweiz tätig sind, nicht in der Schweiz aufgewachsen sind, aber seit 18 bis 28 Jahren in der Schweiz leben. Diese geringe Repräsentation der Schweiz könnte möglicherweise Einfluss auf den länderspezifischen Vergleich genommen haben. Ob die Länderzugehörigkeit auf die Verständlichkeit einwirken kann, wird in Studien kontrovers diskutiert [42, 48]. Demzufolge ist nicht klar erkennbar, wie viel Einfluss unsere Vorgehensweise auf die Verständlichkeitsprüfung und die Übersetzung hatte. Allerdings konnten wir in der Analyse keine sprachlichen Anmerkungen identifizieren, die v. a. von Personen aus der Schweiz, Österreich oder Deutschland gehäuft genannt wurden.

Ein weiteres Kriterium im bewussten Stichprobenplan war das Vorwissen der Interviewpartner*innen in Bezug zur SARA. Ausgeschlossen wurden Interviewpartner*innen, die eine nicht autorisierte deutsche Version der SARA kannten. War das englische Original bekannt oder wurde dieses im klinischen Alltag eingesetzt, wurden die Interviewpartner*innen eingeschlossen. Dies traf auf 9 Interviewpartner*innen zu, wobei 3 davon die englische Version der Skala auch in der Praxis anwendeten. Dadurch könnte man vermuten, dass eine Verständlichkeitsprüfung mit diesen Interviewpartner*innen weniger Erkenntnisse lieferte. In der Analyse stellte sich jedoch heraus, dass aus diesen Interviews aufgrund der ausgezeichneten Englischkenntnisse in Kombination mit dem medizinischen Hintergrundwissen und der praktischen Erfahrung mit der Skala wichtige Aspekte für die Übersetzung genannt wurden. So wurde auf Vorschlag von Interviewpartner*innen 10 bei der Beschreibung des Items 8 die Formulierung „das andere Knie“ zu „das Knie der Gegenseite“ umformuliert. Bei weiteren Übersetzungen von Skalen kann es daher hilfreich sein, Personen in die Verständlichkeitsprüfung mit einzuschließen, die Erfahrungen mit der Skala (in der Originalsprache) mitbringen.

Da die leitliniengerechte Behandlung der motorischen Aspekte der Ataxie nach Klockgether et al. (2018) im interprofessionellen Team erfolgt, rekrutierten wir sowohl Ärtz*innen als auch Physiotherapeut*innen für die Verständlichkeitsprüfung [5]. In den Interviews zeigten sich berufsgruppenspezifische Verständlichkeitsprobleme der Skala: Die Ähnlichkeit von Item 5 bis 8 zu anderen ärztlichen Tests, die in der Untersuchungsroutine Anwendung finden, führte bei ÄrztInnen, die die Originalversion der Skala bisher nicht im klinischen Alltag verwendeten, zu Verständnisschwierigkeiten. Dies könnte eine nicht den Vorgaben entsprechende Durchführung der SARA nach sich ziehen. Eine von der Standardisierung abweichende Durchführung einzelner Items verringert die Reliabilität dieser Items bzw. der gesamten Skala. In Untersuchungen zur Intertester*innen-Reliabilität weisen auch die Items 5 bis 7 geringere ICC-Werte im Vergleich zu anderen Items von unter 0,80 auf [22, 28, 33]. Allerdings wurde die Inter- und Intratester*innen-Reliabilität der SARA in Untersuchungen in verschiedenen Ataxiekohorten mit tendenziell hohen ICC-Werten für den Gesamtscore dargestellt [20, 22, 28–30, 32, 33, 38, 49–52]. Daher dürften die angesprochenen Verständnisprobleme keinen substanziellen Einfluss auf die Verlässlichkeit der Beurteilung haben.

Physiotherapeut*innen hinterfragten vermehrt bewegungs- und haltungsbezogene Ausdrücke wie „Unterstützung durch einen Arm“ oder „normales Sitzen“ und führten an, dass diese Angaben zu allgemein sind und zu nichtreliablen Messergebnissen führen könnten. Die meisten Untersuchungen zur Inter- und Intratester*innen-Reliabilität wurden mit Ärzt*innen durchgeführt [22, 28, 29, 32, 33, 37, 38, 49]. Wir konnten nur 2 Studien finden, die die Reliabilität der SARA durch Physiotherapeut*innen untersuchten: Diese 2 Studien zeigen eine zufriedenstellende Inter- und Intratester*innen-Reliabilität, ähnlich jenen Untersuchungen mit Ärzt*innen. Allerdings basieren diese Studien auf Messungen der Ataxie bei MS als Grunderkrankung [20, 51]. Die Berechnungen basieren außerdem teilweise auf Grundlage eines Videos und nicht auf Grundlage einer weiteren Testung der Proband*innen [51]. Zur Bestätigung einer akzeptablen Inter- und Intratester*innen-Reliabilität der SARA im interprofessionellen Team wären noch weitere Studien v. a. auch mit Personen mit hereditären und sporadischen Formen der Ataxie notwendig.

Einige Interviewpartner*innen empfahlen zur Verbesserung der Verständlichkeit eine Einweisung vor der Verwendung der Skala oder ein ergänzendes Manual. Nähere Erläuterungen zur Art der Anleitung der einzelnen Items der SARA (verbal und/oder nonverbal), eine genauere Beschreibung der Items und der Bewertungsstufe und eine Definition von Schlüsselbegriffen könnten in einem Manual thematisiert werden. Eine Ergänzung zu standardisierten Assessments mit einem Manual erhöht die Reliabilität und Effektivität des Assessments [53]. Somit könnte vermutlich auch die Verständlichkeit optimiert und den Untersucher*innen mehr Sicherheit in der Anwendung der SARA vermitteln werden.

### Praktikabilität der SARA

Bei der Auswertung der Interviews wurden Probleme in der Anwendung der SARA, die in unmittelbarem Zusammenhang mit dem Berufsalltag in Verbindung stehen, genannt. Ärztliche Untersuchungsräume sind häufig zu klein, um, wie in Item 1 angeleitet, eine 10-m-Gehstrecke zu untersuchen. An einigen Arbeitsstellen kann die Anforderung, den Stand (Item 2) auch barfuß zu testen, aufgrund von institutionellen Vorgaben nicht erfüllt werden. Der Positionswechsel für das Testen von nur einem Item (Item 8) wurde außerdem von Physiotherapeut*innen als nicht praktikabel genannt. Inwiefern sich diese Schwierigkeiten auf die Verwendung der SARA im klinischen Gebrauch auswirken, wurde unseres Wissens noch nicht untersucht.

Ein weiterer Aspekt, der von Interviewpartner*innen aufgeworfen wurde, ist die Fokussierung der SARA auf das Testen der ICF-Komponente Körperfunktionen und -strukturen. Vor allem die Items 5 bis 8 (Finger-Folge-Versuch, modifizierter Finger-Nase-Versuch, schnelle alternierende Handbewegungen, Knie-Hacke-Versuch) fokussieren auf Körperfunktionen und -strukturen. In den Items 1 und 4 sind einzelne Aspekte einer Aktivitätsmessung enthalten: So wird in Item 1 eine 10-m-Gehstrecke getestet und in Item 4 die Sprache während der normalen Unterhaltung bewertet. Item 2 (Stehen und Sitzen) sind schwer zuordenbar, enthalten allerdings hauptsächlich Aspekte, die den neuromuskuloskeletalen und bewegungsbezogenen Funktionen zuzuordnen sind. Eine gesamtheitliche Erfassung des funktionalen Gesundheitszustandes ist, wie die Interviewpartner*innen anmerkten, mit der SARA nicht möglich, da Aktivität und v. a. Partizipation nicht erhoben werden.

Die interviewten Personen hatten im Praxisalltag mit verschiedenen Formen der Ataxie Erfahrung. In diesem Zusammenhang tauchten in den Interviews Überlegungen auf, ob die SARA bei symptomatischen Ataxien, die teilweise zusätzliche neurologische Symptome wie Tonuserhöhungen oder Hemiparesen aufweisen, ähnlich gute psychometrische Eigenschaften aufweist wie bei hereditären oder idiopathischen Ataxien. Speziell wurde die Kalkulation des Durchschnittscores beider Seiten, der bei Item 5 bis 8 zu notieren ist, beim Testen von Personen mit Hemiparese hinterfragt. Wir konnten 2 Studien finden, die die SARA bei Personen nach Schlaganfall (in der akuten und subakuten Phase) untersuchten [32, 34], und 2 weitere bei Kindern mit Gehirntumoren [30, 54]. Außerdem wurden bei Salci et al. (2017) die Validität und Reliabilität der türkischen Version der Skala bei ataktischen Personen mit MS untersucht [20]. Keine der genannten Studien publizierte Daten zur Tonussituation der getesteten Personen. Bei Kim et al. (2011) waren 53 % (32 Personen) der inkludierten Personen mit einer rechts- oder linkshirnigen Läsion beschrieben, eine detaillierte Analyse im Vergleich zu den bilateral betroffenen Personen fand aber nicht statt [32]. Panzeri et al. (2020) stellten den Kindern mit zusätzlicher Hemiparese Kinder mit isolierter Ataxie gegenüber und fanden statistisch signifikante Gruppenunterschiede im Gesamtscore und bei einzelnen Items [54]. Für die Analyse der Items 5 bis 8 wurde jede Körperseite für sich analysiert und nicht wie in der Originalversion der SARA ein Durchschnittsscore berechnet. Aufgrund der Studie von Panzeri et al. (2020) kann vermutet werden, dass die Hemiparese die Bewertung von Ataxiezeichen beeinflusst [54]. Inwieweit dies mit begleitenden Tonuserhöhungen einhergeht, kann nicht abschließend beurteilt werden.

### Limitationen

Die Übersetzung der Skala fand nach internationalen Richtlinien statt und hatte zum Ziel, die Skala für den gesamten deutschsprachigen Raum in der Sprache der klinischen Anwender*innen verfügbar zu machen. In der Rekrutierung für die Interviews konnten im Verhältnis weniger Personen aus Österreich bzw. der Schweiz im Vergleich zu Deutschland rekrutiert werden. Inwieweit Deutschland, Schweiz und Österreich bezüglich standardisierter Assessments eine länderspezifische oder regionale Anpassung benötigen, ist unklar. Wir konnten in unseren Interviews keine länderspezifischen Unterschiede feststellen und bestätigen damit entsprechende Ergebnisse aus der Literatur [42].

Für die Verständlichkeitstestung im Rahmen einer kulturellen Übersetzung wird empfohlen, 30 bis 40 Personen zu rekrutieren [18]. Betreffend kognitiver Pretests wird angeraten, 5 bis 30 Interviews durchzuführen [40]. Wir rekrutierten 15 Personen und konnten damit unsere Forschungsfragen beantworten. Die Interviews wiesen inhaltliche Wiederholungen auf. Andere Übersetzungsarbeiten für die SARA führten entweder keine Verständlichkeitstestungen [55–58] oder Verständlichkeitstestungen mit weniger Personen durch [20, 59]. Kognitive Interviews wie in der vorliegenden Studie wurden bei anderen Übersetzungen der SARA nicht durchgeführt.

Bei der Durchführung von kognitiven Interviews wird ein iteratives Vorgehen mit bis zu 4 Testrunden empfohlen [40, 60]. Die von uns herangezogenen Richtlinien [18, 47] für die Übersetzung von Assessments fordern diese iterativen Testrunden nicht ein. Wir überprüften in einer Feedbackschleife die Verständlichkeit der durch die Interviews vorgenommenen Änderungen der Skala. Wir können jedoch nicht ausschließen, dass weitere Iterationen zusätzliche Aspekte der Verständlichkeit hervorgebracht hätten.

## Kernbotschaft

In dieser Arbeit wurde die englischsprachige „Scale for the assessment and rating of ataxia“ (SARA) nach internationalen Richtlinien ins Deutsche übersetzt und von der Mitautorin der Originalskala autorisiert. Aufgabe von zukünftigen Forschungsarbeiten wird die Untersuchung der psychometrischen Eigenschaften der deutschen Version der SARA sein.

Die ausführlich durchgeführte Verständlichkeitstestung führte nicht nur zu sprachlichen Anpassungen in der Übersetzung, sondern zeigte allgemeine Anwendungsschwierigkeiten der Skala auf. Diese könnten bei weiteren Entwicklungen berücksichtigt werden. Insofern ist eine ausführliche Verständlichkeitstestung nicht nur für die sprachliche Anpassung hilfreich, sondern könnte auch richtungsweisend für die Weiterentwicklung einer Skala bzw. für die Entwicklung neuer Skalen sein. Unsere Erfahrungen zeigen, dass im deutschsprachigen Raum eine Rekrutierung aus Deutschland, der Schweiz und aus Österreich nicht unbedingt notwendig ist.

Bei weiteren Untersuchungen zur Konstrukt- und Kriteriumsvalidität sollten Personen mit symptomatischen Ataxien besondere Beachtung finden. In bisherigen Untersuchungen zu den psychometrischen Eigenschaften der Skala wendeten Ärzt*innen die SARA an; die Behandlung der Ataxie erfolgt laut Leitlinien aber im interprofessionellen Team. Daher wäre es wünschenswert, weitere Berufsgruppen in die Überprüfung der Inter- und Intratester*innen-Reliabilität einzubinden.

### Supplementary Information





