# Supplementary material for: Scale for the assessment and rating of ataxia (SARA): translation and cultural adaptation to German-speaking areas
Source: Wien Med Wochenschr. 2023 Apr 24;174(5-6):111–22. [Article in German] doi: 10.1007/s10354-023-01014-8 (PMC10959797; doi:10.1007/s10354-023-01014-8)
Supplement: Supplementary file 1 [file 10354_2023_1014_MOESM1_ESM.docx]

**Supplemental File 1: Übersetzungsprozess - Versionen der SARA**

Grau markierte Stellen wurden nach dem Expert*innenkomitee bzw. nach den Interviews verändert.

|  | **Synthese** | **Arbeitsversion** | **deutsche Version** |
| --- | --- | --- | --- |
| Daten | UntersucherIn: ________Datum:________PatientIn/ Person:________ | UntersucherIn: ________Datum:________PatientIn/ Person:________ | Untersucher*in: ________Datum:________PatientI*i/ Person:________ |
| Überschrift | Skala für Assessment und Rating von Ataxie (SARA) | Skala für das Assessment und Rating von Ataxie (SARA) | Skala für das Assessment und Rating von Ataxie (SARA) |
| Item 1 | 1) Gang | 1) Gang | 1) Gang |
| Anleitung | Die Person wird gebeten (1) parallel, in einem sicheren Abstand zu einer Wand, zu gehen, dann eine halbe Drehung zu machen (Umdrehen, um in die andere Richtung zu gehen) und (2) im Tandemgang (Fersen an Zehen) ohne Unterstützung zu gehen. | Die Person wird gebeten  (1) in einem sicheren Abstand parallel zu einer Wand zu gehen, dann eine halbe Drehung zu machen, um in die andere Richtung zu gehen und (2) ohne Unterstützung im Tandemgang (Fersen an Zehen) zurück zu gehen. | Die Person wird gebeten  (1) in einem sicheren Abstand entlang einer Wand zu gehen, dann eine halbe Drehung zu machen, um in die andere Richtung zu gehen und (2) ohne Unterstützung im Tandemgang (Fersen an Zehen) zu gehen. |
| Bewertungsstufen | 0 Normal, keine Schwierigkeiten beim Gehen, Umdrehen und im Tandemgang (ein Fehltritt ist erlaubt) | 0 Normal, keine Schwierigkeiten beim Gehen, Umdrehen und im Tandemgang (ein Fehltritt ist erlaubt) | 0 Normal, keine Schwierigkeiten beim Gehen, beim Umdrehen und beim Tandemgang (ein Ausweichschritt ist erlaubt) |
|  | 1 Leichte Schwierigkeiten, nur sichtbar wenn 10 aufeinanderfolgende Schritte im Tandemgang gemacht werden | 1 Leichte Schwierigkeiten, nur sichtbar in 10 aufeinanderfolgenden Schritten im Tandemgang | 1 Leichte Schwierigkeiten, nur sichtbar bei 10 aufeinanderfolgenden Schritten im Tandemgang |
|  | 2 Eindeutig abweichend, Tandemgang > 10 Schritte nicht möglich | 2 Eindeutig auffällig, Tandemgang > 10 Schritte nicht möglich | 2 Eindeutig auffällig, Tandemgang > 10 Schritte nicht möglich |
|  | 3 Beträchtliches Wanken, Schwierigkeiten bei der halben Drehung aber ohne Unterstützung möglich | 3 Beträchtliches Wanken, Schwierigkeiten bei der halben Drehung, aber ohne Unterstützung möglich | 3 Beträchtliches Wanken, Schwierigkeiten bei der halben Drehung, aber ohne Unterstützung möglich |
|  | 4 Deutliches Wanken, teilweise Stütz an der Wand notwendig | 4 Deutliches Wanken, teilweise Abstützen an der Wand erforderlich | 4 Deutliches Wanken, intermittierendes Abstützen an der Wand erforderlich |
|  | 5 Stark ausgeprägtes Wanken, dauerhafte Verwendung eines Gehstock oder minimale Unterstützung durch einen Arm notwendig | 5 Stark ausgeprägtes Wanken, dauerhafte Verwendung eines Gehstocks oder minimale Unterstützung durch einen Arm erforderlich | 5 Stark ausgeprägtes Wanken, konstante Verwendung eines Gehstocks oder minimale Unterstützung durch einen Arm erforderlich |
|  | 6 Gehen > 10m nur mit viel Unterstützung (zwei spezielle Gehstöcke, Rollator/Rollmobil oder unterstützende Person) | 6 Gehen > 10m nur mit viel Unterstützung (zwei spezielle Gehstöcke, Rollator/Rollmobil oder unterstützende Person) | 6 Gehen > 10m nur mit viel Unterstützung (zwei spezielle Gehstöcke, Rollator/Rollmobil oder unterstützende Person) |
|  | 7 Gehen < 10m nur mit viel Unterstützung (zwei spezielle Gehstöcke, Rollator/Rollmobil oder unterstützende Person) | 7 Gehen < 10m nur mit viel Unterstützung (zwei spezielle Gehstöcke, Rollator/Rollmobil oder unterstützende Person) | 7 Gehen < 10m nur mit viel Unterstützung (zwei spezielle Gehstöcke, Rollator/Rollmobil oder unterstützende Person) |
|  | 8 Gehen nicht möglich, auch nicht mit Unterstützung | 8 Gehen auch mit Unterstützung nicht möglich | 8 Gehen auch mit Unterstützung nicht möglich |
|  | Punkte | Punkte | Punkte |
| Item 2 | 2) Stand | 2) Stand | 2) Stand |
| Anleitung | Die Person wird gebeten zu stehen: (1) in einer natürlichen Position, (2) mit geschlossenen, parallel zueinanderstehenden Füßen (Großzehen berühren einander) und  (3) im Tandemstand (beide Füße in einer Linie, kein Abstand zwischen Ferse und Zehen) | Die Person trägt keine Schuhe und die Augen sind geöffnet. Die Person wird gebeten zu stehen: (1) in einer natürlichen Position, (2) mit geschlossenen, parallel zueinanderstehenden Füßen (Großzehen berühren einander) und  (3) im Tandemstand (beide Füße in einer Linie, kein Abstand zwischen Ferse und Zehen).  Für jede Übung sind 3 Versuche erlaubt. Der beste Versuch wird gewertet. | Die Person trägt keine Schuhe und die Augen sind geöffnet. Die Person wird gebeten zu stehen: (1) in einer natürlichen Position, (2) mit geschlossenen, parallel zueinanderstehenden Füßen (Großzehen berühren einander) und  (3) im Tandemstand (beide Füße in einer Linie, kein Abstand zwischen Ferse und Zehen).  Bei jeder Aufgabe sind 3 Versuche erlaubt. Der beste Versuch wird gewertet. |
| Bewertungsstufen | 0 Normal, Tandemstand > 10 Sekunden möglich | 0 Normal, Tandemstand > 10 Sekunden möglich | 0 Normal, Tandemstand > 10 Sekunden (Sek.) möglich |
|  | 1 Stehen mit geschlossenen Füßen ohne zu Wanken möglich, aber nicht im Tandemstand für > 10 Sekunden | 1 Stehen mit geschlossenen Füßen ohne Schwanken für > 10 Sekunden möglich, aber nicht im Tandemstand | 1 Stehen mit geschlossenen Füßen ohne Schwanken möglich, aber nicht im Tandemstand für > 10 Sek., |
|  | 2 Stehen mit geschlossenen Füßen für > 10 Sekunden möglich, aber nur mit Wanken | 2 Stehen mit geschlossenen Füßen für > 10 Sekunden möglich, aber nur mit Schwanken | 2 Stehen mit geschlossenen Füßen für > 10 Sek. möglich, aber nur mit Schwanken |
|  | 3 Stehen in einer natürlichen Position ohne Unterstützung für > 10 Sekunden möglich, aber nicht mit geschlossenen Füßen | 3 Stehen in einer natürlichen Position ohne Unterstützung für > 10 Sekunden möglich, aber nicht mit geschlossenen Füßen | 3 Stehen in einer natürlichen Position ohne Unterstützung für > 10 Sek. möglich, aber nicht mit geschlossenen Füßen |
|  | 4 Stehen in einer natürlichen Position für >10 Sekunden möglich, aber nur mit teilweiser Unterstützung | 4 Stehen in einer natürlichen Position nur mit teilweiser Unterstützung für > 10 Sekunden möglich | 4 Stehen in einer natürlichen Position nur mit intermittierender Unterstützung für > 10 Sek. möglich |
|  | 5 Stehen für > 10 Sekunden in einer natürlichen Position nur mit dauerhafter Unterstützung durch einen Arm | 5 Stehen in einer natürlichen Position nur mit dauerhafter Unterstützung durch einen Arm für > 10 Sekunden möglich | 5 Stehen in einer natürlichen Position nur mit konstanter Unterstützung durch einen Arm für > 10 Sek. möglich |
|  | 6 Stehen für > 10 Sekunden auch mit dauerhafter Unterstützung eines Armes nicht möglich | 6 Stehen auch mit dauerhafter Unterstützung durch einen Arm für > 10 Sekunden nicht möglich | 6 Stehen auch mit konstanter Unterstützung durch einen Arm für > 10 Sek. nicht möglich |
|  | Punkte | Punkte | Punkte |
| Item 3 | 3) Sitz | 3) Sitzen | 3) Sitzen |
| Anleitung | Die Person wird gebeten, ohne Bodenkontakt auf einer Therapieliege zu sitzen, die Augen sind geöffnet und die Arme sind nach vorne ausgestreckt. | Die Person wird gebeten, auf einer Untersuchungsliege ohne Bodenkontakt der Füße zu Sitzen. Die Augen sind geöffnet und die Arme sind nach vorne ausgestreckt. | Die Person wird gebeten, auf einer Untersuchungsliege ohne Bodenkontakt der Füße zu sitzen. Die Augen sind geöffnet und die Arme sind nach vorne ausgestreckt. |
| Bewertungsstufen | 0 Normal, Sitzen > 10 Sekunden ohne Schwierigkeiten möglich | 0 Normal, sitzen ohne Schwierigkeiten für > 10 Sekunden möglich | 0 Normal, Sitzen ohne Schwierigkeiten für > 10 Sek. möglich |
|  | 1 Leichte Schwierigkeiten, teilweise Wanken | 1 Leichte Schwierigkeiten, teilweise Schwanken | 1 Leichte Schwierigkeiten, intermittierendes Schwanken |
|  | 2 Dauerhaftes Wanken, aber Sitzen > 10 Sekunden ohne Unterstützung möglich | 2 Dauerhaftes Schwanken, aber sitzen ohne Unterstützung > 10 Sekunden möglich | 2 Konstantes Schwanken, aber Sitzen ohne Unterstützung > 10 Sek. möglich |
|  | 3 Sitzen für > 10 Sekunden, mit teilweiser Unterstützung, möglich | 3 Sitzen nur mit teilweiser Unterstützung für > 10 Sekunden möglich | 3 Sitzen nur mit intermittierender Unterstützung für > 10 Sek. möglich |
|  | 4 Nicht möglich, ohne dauerhafte Unterstützung, für > 10 Sekunden zu sitzen | 4 Sitzen ohne dauerhafte Unterstützung für > 10 Sekunden nicht möglich | 4 Sitzen ohne konstante Unterstützung für > 10 Sek. nicht möglich |
|  | Punkte | Punkte | Punkte |
| Item 4 | 4) Sprechen | 4) Sprechen/Sprechstörung | 4) Sprechstörung |
| Anleitung | Das Sprechen wird während der normalen Unterhaltung beurteilt. | Das Sprechen wird während der normalen Unterhaltung bewertet. | Das Sprechen wird während der normalen Unterhaltung bewertet. |
| Bewertungsstufen | 0 Normal | 0 Normal | 0 Normal |
|  | 1 Hinweis auf eine Sprechstörung | 1 Hinweis auf eine Sprechstörung | 1 Andeutung einer Sprechstörung |
|  | 2 Beeinträchtigtes Sprechen, aber leicht verständlich | 2 Beeinträchtigtes Sprechen, aber leicht verständlich | 2 Beeinträchtigtes Sprechen, aber leicht verständlich |
|  | 3 Vereinzelte Wörter schwer verständlich | 3 Vereinzelte Wörter schwer verständlich | 3 Einzelne Wörter schwer verständlich |
|  | 4 Viele Wörter schwer verständlich | 4 Viele Wörter schwer verständlich | 4 Viele Wörter schwer verständlich |
|  | 5 Nur einzelne Wörter verständlich | 5 Nur einzelne Wörter verständlich | 5 Nur einzelne Wörter verständlich |
|  | 6 Sprechen unverständlich/Anarthrie | 6 Sprechen unverständlich/Anarthrie | 6 Sprechen unverständlich/Anarthrie |
|  | Punkte | Punkte | Punkte |
| Item 5 | 5) Finger-Finger Versuch | 5) Finger-Folge Versuch | 5) Finger-Folge Versuch |
| Anleitung | Für jede Seite separat bewerten Die Person sitzt bequem. Bei Bedarf können Füße oder Rumpf unterstützt werden. Der Untersucher sitzt vor der Person und führt 5 aufeinanderfolgende, plötzliche und schnelle Bewegungen in unvorhersehbaren Richtungen in der Frontalebene, innerhalb cirka 50% der Reichweite der Person, durch. Die Bewegungen haben eine Amplitude von 30 cm und eine Frequenz von 1 Bewegung alle 2 Sekunden. Die Person soll den Bewegungen mit dem Zeigefinger so schnell und genau wie möglich folgen. Die durchschnittliche Ausführung der letzten drei Bewegungen wird beurteilt. | Für jede Seite einzeln werten Die Person sitzt bequem. Bei Bedarf können Füße oder Rumpf unterstützt werden. Die/Der Untersuchende sitzt vor der Person und führt 5 aufeinanderfolgende, plötzliche und schnelle Zeigebewegungen durch. Diese finden in unvorhersehbaren Richtungen in der Frontalebene, innerhalb circa 50% der Reichweite der Person statt. Die Bewegungen haben eine Amplitude von 30 cm und eine Frequenz von 1 Bewegung/2 Sekunden. Die Person wird gebeten den Bewegungen mit dem Zeigefinger so schnell und genau wie möglich zu folgen. Die durchschnittliche Ausführung der letzten drei Bewegungen wird bewertet. | Für jede Seite einzeln werten Die Person sitzt bequem, eventuell Füße und Rumpf unterstützen. Die/der Untersuchende sitzt vor der Person und führt 5 aufeinanderfolgende plötzliche und schnelle Zeigebewegungen durch. Diese finden in unvorhersehbaren Richtungen in der Frontalebene innerhalb ca. 50% der Reichweite der Person statt. Die Bewegungen haben eine Amplitude von 30 cm und eine Frequenz von 1 Bewegung/2 Sek. Die Person wird gebeten, den Bewegungen mit dem Zeigefinger so schnell und genau wie möglich zu folgen. Die durchschnittliche Ausführung der letzten 3 Bewegungen wird bewertet. |
| Bewertungsstufen | 0 Keine Dysmetrie | 0 Keine Dysmetrie | 0 Keine Dysmetrie |
|  | 1 Dysmetrie, unter-/überschießen des Ziel <5cm | 1 Dysmetrie, Unter-/Überschießen des Ziels < 5cm | 1 Dysmetrie, Unter-/Überschießen des Ziels < 5cm |
|  | 2 Dysmetrie, unter-/überschießen des Ziel <15cm | 2 Dysmetrie, Unter-/Überschießen des Ziels < 15cm | 2 Dysmetrie, Unter-/Überschießen des Ziels < 15cm |
|  | 3 Dysmetrie, unter-/überschießen des Ziel >15cm | 3 Dysmetrie, Unter-/Überschießen des Ziels > 15cm | 3 Dysmetrie, Unter-/Überschießen des Ziels > 15cm |
|  | 4 Nicht möglich 5 Zeigebewegungen durchzuführen | 4 Durchführen von 5 Zeigebewegungen nicht möglich | 4 Durchführen von 5 Zeigebewegungen nicht möglich |
|  | Punkte Rechts Links | Punkte Rechts Links | Punkte Rechts Links |
|  | Durchschnitt beider Seiten (R+L)/2 | Punkte Durchschnitt beider Seiten | Punkte Mittelwert beider Seiten |
|  |  | (R+L)/2 | (R+L)/2 |
| Item 6 | 6) Finger-Nase Test | 6) Finger-Nase Versuch | 6) modifizierter Finger-Nase Versuch |
| Anleitung | Für jede Seite separat bewerten Die Person sitzt bequem. Bei Bedarf können Füße oder Rumpf unterstützt werden. Die Person wird gebeten, mehrmals den Zeigefinger von der eigenen Nase zum Finger des Untersuchenden zu bewegen. Dieser Finger befindet sich in cirka 90% der Reichweite der Person. Die Bewegungen werden in moderater Geschwindigkeit durchgeführt. Die durchschnittliche Ausführung der Bewegungen wird entsprechend des kinetischen Tremors beurteilt. | Für jede Seite einzeln werten Die Person sitzt bequem. Bei Bedarf können Füße oder Rumpf unterstützt werden. Der/die Untersuchende hält seinen Finger in circa 90% Reichweite vor der Person. Die Person wird gebeten, mehrmals den Zeigefinger von der eigenen Nase zum Finger der/des Untersuchenden zu bewegen. Die Bewegungen werden in moderater Geschwindigkeit durchgeführt. Die durchschnittliche Ausführung der Bewegungen wird entsprechend der Ausprägung des kinetischen Tremors bewertet. | Für jede Seite einzeln werten Die Person sitzt bequem, eventuell Füße und Rumpf unterstützen. Die/der Untersuchende hält seinen Finger in ca. 90% Reichweite vor der Person. Die Person wird gebeten, mehrmals den Zeigefinger von der eigenen Nase zum Finger der/des Untersuchenden zu bewegen. Die Bewegungen werden in moderater Geschwindigkeit durchgeführt. Die durchschnittliche Ausführung der Bewegungen wird entsprechend der Ausprägung des kinetischen Tremors bewertet. |
| Bewertungsstufen | 0 Kein Tremor | 0 Kein Tremor | 0 Kein Tremor |
|  | 1 Tremor mit einer Amplitude von < 2 cm | 1 Tremor mit einer Amplitude von < 2cm | 1 Tremor mit einer Amplitude von < 2cm |
|  | 2 Tremor mit einer Amplitude von < 5 cm | 2 Tremor mit einer Amplitude von < 5cm | 2 Tremor mit einer Amplitude von < 5cm |
|  | 3 Tremor mit einer Amplitude von > 5 cm | 3 Tremor mit einer Amplitude von > 5cm | 3 Tremor mit einer Amplitude von > 5cm |
|  | 4 Nicht möglich 5 Zeigebewegungen durchzuführen | 4 Durchführen von 5 Zeigebewegungen nicht möglich | 4 Durchführen von 5 Zeigebewegungen nicht möglich |
|  | Punkte Rechts Links | Punkte Rechts Links | Punkte Rechts Links |
|  | Durchschnitt beider Seiten (R+L)/2 | Punkte Durchschnitt beider Seiten | Punkte Mittelwert beider Seiten |
|  |  | (R+L)/2 | (R+L)/2 |
| Item 7 | 7) Schnelle abwechselnde Handbewegungen | 7) Schnelle wechselnde Handbewegungen | 7) Schnelle alternierende Handbewegungen |
| Anleitung | Für jede Seite separat bewerten Die Person sitzt bequem. Bei Bedarf können Füße oder Rumpf unterstützt werden. Die Person wird gebeten, 10 Zyklen von repetitiv abwechselnder Pro- und Supination der Hand auf dem Oberschenkel, so schnell und genau wie möglich, durchzuführen. Die Bewegung wird, mit einer Geschwindigkeit von 10 Zyklen innerhalb von 7 Sekunden, vorgezeigt. Die exakten Zeiten für die Bewegungsausführung müssen gemessen werden. | Für jede Seite einzeln werten Die Person sitzt bequem. Bei Bedarf können Füße oder Rumpf unterstützt werden. Die Person wird gebeten, 10 Zyklen von repetitiv abwechselnder Pro- und Supination der Hand auf dem Oberschenkel, so schnell und genau wie möglich durchzuführen. Die Bewegung wird mit einer Geschwindigkeit von circa 10 Zyklen/7 Sekunden vorgezeigt. Die exakten Zeiten für die Bewegungsausführung der Person müssen gemessen werden. | Für jede Seite einzeln werten Die Person sitzt bequem, eventuell Füße und Rumpf unterstützen. Die Person wird gebeten, 10 Wiederholungen von alternierenden Pro- und Supinationen der Hand auf dem Oberschenkel, so schnell und so genau wie möglich durchzuführen. Die Bewegung wird mit einer Geschwindigkeit von ca. 10 Wiederholungen/7 Sek. vorgezeigt. Die exakten Zeiten für die Bewegungsausführung der Person müssen gemessen werden. |
| Bewertungsstufen | 0 Normal, keine Unregelmäßigkeiten (durchgeführt <10 Sekunden) | 0 Normal, keine Unregelmäßigkeiten (durchgeführt in < 10 Sekunden) | 0 Normal, keine Unregelmäßigkeiten (durchgeführt in < 10 Sek.) |
|  | 1 Leicht unregelmäßig (durchgeführt <10 Sekunden) | 1 Leicht unregelmäßig (durchgeführt in < 10 Sekunden) | 1 Leicht unregelmäßig (durchgeführt in < 10 Sek.) |
|  | 2 Deutlich unregelmäßig, einzelne Bewegungen schwer zu erkennen oder unterbrochen, aber durchgeführt <10 Sekunden | 2 Deutlich unregelmäßig, einzelne Bewegungen schwer zu erkennen oder unterbrochen (durchgeführt in < 10 Sekunden) | 2 Deutlich unregelmäßig, einzelne Bewegungen schwer zu unterscheiden oder unterbrochen (durchgeführt in < 10 Sek.) |
|  | 3 Sehr unregelmäßig, einzelne Bewegungen schwer zu erkennen oder unterbrochen, durchgeführt >10 Sekunden | 3 Sehr unregelmäßig, einzelne Bewegungen schwer zu erkennen oder unterbrochen (durchgeführt in > 10 Sekunden) | 3 Sehr unregelmäßig, einzelne Bewegungen schwer zu unterscheiden oder unterbrochen (durchgeführt in > 10 Sek.) |
|  | 4 Nicht möglich 10 Zyklen durchzuführen | 4 Durchführen von 10 Zyklen nicht möglich | 4 Durchführen von 10 Wiederholungen nicht möglich |
|  | Punkte Rechts Links | Punkte Rechts Links | Punkte Rechts Links |
|  | Durchschnitt beider Seiten (R+L)/2 | Punkte Durchschnitt beider Seiten | Punkte Mittelwert beider Seiten |
|  |  | (R+L)/2 | (R+L)/2 |
| Item 8 | 8) Knie-Hacke Versuch | 8) Knie-Hacke Versuch | 8) Knie-Hacke Versuch |
| Anleitung | Für jede Seite separat bewerten Die Person liegt auf einer Therapieliege, ohne die Beine zu sehen. Sie wird gebeten, ein Bein anzuheben, mit der Ferse das andere Knie zu berühren und entlang des Schienbeins zum Knöchel zu gleiten und anschließend das Bein zurück auf die Liege zu legen. Diese Aufgabe wird 3 Mal wiederholt. Die Gleitbewegung sollte innerhalb 1 Sekunde durchgeführt werden. Wird die Bewegung ohne Berührung des Schienbeins alle 3 Mal durchgeführt, so wird dies mit 4 beurteilt. | Für jede Seite einzeln werten  Die Person liegt auf einer Untersuchungsliege, ohne auf die Beine zu sehen. Sie wird gebeten, ein Bein anzuheben, mit der Ferse das andere Knie zu berühren und entlang des Schienbeins zum Knöchel zu gleiten und anschließend das Bein zurück auf die Liege zu legen. Diese Aufgabe wird 3 Mal wiederholt. Jede Gleitbewegung sollte innerhalb 1 Sekunde durchgeführt werden. Wird die Gleitbewegung in allen 3 Wiederholungen ohne Kontakt der Ferse zum Schienbein durchgeführt, so wird dies mit 4 bewertet. | Für jede Seite einzeln werten  Die Person liegt auf einer Untersuchungsliege, ohne auf die Beine zu sehen. Sie wird gebeten, ein Bein anzuheben, mit der Ferse das Knie der Gegenseite zu berühren und entlang des Schienbeins zum Knöchel zu gleiten und anschließend das Bein zurück auf die Liege zu legen. Diese Aufgabe wird 3 Mal wiederholt. Jede Gleitbewegung sollte innerhalb 1 Sek. durchgeführt werden. Wird die Gleitbewegung bei allen 3 Wiederholungen ohne Kontakt der Ferse zum Schienbein durchgeführt, so wird dies mit 4 bewertet. |
| Bewertungsstufen | 0 Normal | 0 Normal | 0 Normal |
|  | 1 Leicht abnormal, Kontakt zum Schienbein vorhanden | 1 Leicht auffällig, Kontakt zum Schienbein bleibt erhalten | 1 Leicht auffällig, Kontakt zum Schienbein bleibt erhalten |
|  | 2 Deutlich abnormal, Kontakt zum Schienbein geht bis zu 3 Mal innerhalb der 3 Durchläufe verloren | 2 Deutlich auffällig, Kontakt zum Schienbein geht bis zu 3 Mal über 3 Wiederholungen verloren | 2 Deutlich auffällig, Kontakt zum Schienbein geht bis zu 3 Mal über alle 3 Wiederholungen verloren |
|  | 3 Stark abnormal, Kontakt zum Schienbein geht 4 Mal oder öfter innerhalb der 3 Durchläufe verloren | 3 Stark auffällig, Kontakt zum Schienbein geht 4 Mal oder öfter über 3 Wiederholungen verloren | 3 Stark auffällig, Kontakt zum Schienbein geht 4 Mal oder öfter über alle 3 Wiederholungen verloren |
|  | 4 Nicht möglich die Aufgabe durchzuführen | 4 Durchführen der Aufgabe nicht möglich | 4 Durchführen der Aufgabe nicht möglich |
|  | Punkte Rechts Links | Punkte Rechts Links | Punkte Rechts Links |
|  | Durchschnitt beider Seiten (R+L)/2 | Punkte Durchschnitt beider Seiten | Punkte Mittelwert beider Seiten |
|  |  | (R+L)/2 | (R+L)/2 |
| Nachsatz |  |  | SARA-Score berechnet sich aus der Summe aller grau hinterlegten Felder (Spanne 0-40; siehe Schmitz-Hübsch et al. 2006. Scale for the assessment and rating of ataxia: development of a new clinical scale); kulturelle Anpassung und Übersetzung ins Deutsche: …… |
