# Supplementary material for: Scale for the assessment and rating of ataxia (SARA): translation and cultural adaptation to German-speaking areas
Source: Wien Med Wochenschr. 2023 Apr 24;174(5-6):111–22. [Article in German] doi: 10.1007/s10354-023-01014-8 (PMC10959797; doi:10.1007/s10354-023-01014-8)
Supplement: Supplementary file 2 [file 10354_2023_1014_MOESM2_ESM.docx]

Untersucher*in : __________________Datum: ___________________Patient*in/Person: __________________

**Skala für das Assessment und Rating von Ataxie (SARA)**

| **1) Gang**  Die Person wird gebeten  (1) in einem sicheren Abstand entlang einer Wand zu gehen, dann eine halbe Drehung zu machen, um in die andere Richtung zu gehen und  (2) ohne Unterstützung im Tandemgang (Fersen an Zehen) zu gehen.   1. **Normal, keine Schwierigkeiten beim Gehen, beim Umdrehen und beim Tandemgang (ein Ausweichschritt ist erlaubt)** 2. **Leichte Schwierigkeiten, nur sichtbar bei 10 aufeinanderfolgenden Schritten im Tandemgang** 3. **Eindeutig auffällig, Tandemgang > 10 Schritte nicht möglich** 4. **Beträchtliches Wanken, Schwierigkeiten bei der halben Drehung, aber ohne Unterstützung möglich** 5. **Deutliches Wanken, intermittierendes Abstützen an der Wand erforderlich** 6. **Stark ausgeprägtes Wanken, konstante Verwendung eines Gehstocks oder minimale Unterstützung durch einen Arm erforderlich** 7. **Gehen > 10m nur mit viel Unterstützung (zwei spezielle Gehstöcke, Rollator/Rollmobil oder unterstützende Person)** 8. **Gehen < 10m nur mit viel Unterstützung (zwei spezielle Gehstöcke, Rollator/Rollmobil oder unterstützende Person)** 9. **Gehen auch mit Unterstützung nicht möglich** | | **2) Stand**  Die Person trägt keine Schuhe und die Augen sind geöffnet. Die Person wird gebeten zu stehen:  (1) in einer natürlichen Position,  (2) mit geschlossenen, parallel zueinanderstehenden Füßen (Großzehen berühren einander) und  (3) im Tandemstand (beide Füße in einer Linie, kein Abstand zwischen Ferse und Zehen).  Bei jeder Aufgabe sind 3 Versuche erlaubt. Der beste Versuch wird gewertet.   1. **Normal, Tandemstand > 10 Sekunden (Sek.) möglich** 2. **Stehen mit geschlossenen Füßen ohne Schwanken möglich, aber nicht im Tandemstand für > 10 Sek.** 3. **Stehen mit geschlossenen Füßen für > 10 Sek. möglich, aber nur mit Schwanken** 4. **Stehen in einer natürlichen Position ohne Unterstützung für > 10 Sek. möglich, aber nicht mit geschlossenen Füßen** 5. **Stehen in einer natürlichen Position nur mit intermittierender Unterstützung für > 10 Sek. möglich** 6. **Stehen in einer natürlichen Position nur mit konstanter Unterstützung durch einen Arm für > 10 Sek. möglich** 7. **Stehen auch mit konstanter Unterstützung durch einen Arm für > 10 Sek. nicht möglich** | |
| --- | --- | --- | --- |
| **Punkte** |  | **Punkte** |  |
| **3) Sitzen**  Die Person wird gebeten, auf einer Untersuchungsliege ohne Bodenkontakt der Füße zu sitzen. Die Augen sind geöffnet und die Arme sind nach vorne ausgestreckt.   1. **Normal, Sitzen ohne Schwierigkeiten für > 10 Sek. möglich** 2. **Leichte Schwierigkeiten, intermittierendes Schwanken** 3. **Konstantes Schwanken, aber Sitzen ohne Unterstützung > 10 Sek. möglich** 4. **Sitzen nur mit intermittierender Unterstützung für  > 10 Sek. möglich** 5. **Sitzen ohne konstante Unterstützung für > 10 Sek. nicht möglich** | | **4) Sprechstörung**  Das Sprechen wird während der normalen Unterhaltung bewertet.   1. **Normal** 2. **Andeutung einer Sprechstörung** 3. **Beeinträchtigtes Sprechen, aber leicht verständlich** 4. **Einzelne Wörter schwer verständlich** 5. **Viele Wörter schwer verständlich** 6. **Nur einzelne Wörter verständlich** 7. **Sprechen unverständlich/Anarthrie** | |
| **Punkte** |  | **Punkte** |  |

UntersucherIn : __________________Datum: ___________________PatientIn/Person: ___________________

| **5) Finger-Folge Versuch**  **Für jede Seite einzeln werten**  Die Person sitzt bequem, eventuell Füße und Rumpf unterstützen. Die/der Untersuchende sitzt vor der Person und führt 5 aufeinanderfolgende plötzliche und schnelle Zeigebewegungen durch. Diese finden in unvorhersehbaren Richtungen in der Frontalebene innerhalb ca. 50% der Reichweite der Person statt. Die Bewegungen haben eine Amplitude von 30 cm und eine Frequenz von 1 Bewegung/2 Sek. Die Person wird gebeten, den Bewegungen mit dem Zeigefinger so schnell und genau wie möglich zu folgen. Die durchschnittliche Ausführung der letzten 3 Bewegungen wird bewertet.   1. **Keine Dysmetrie** 2. **Dysmetrie, Unter-/Überschießen des Ziels < 5cm** 3. **Dysmetrie, Unter-/Überschießen des Ziels < 15cm** 4. **Dysmetrie, Unter-/Überschießen des Ziels > 15cm** 5. **Durchführen von 5 Zeigebewegungen nicht möglich** | | | **6) modifizierter Finger-Nase Versuch**  **Für jede Seite einzeln werten**  Die Person sitzt bequem, eventuell Füße und Rumpf unterstützen. Die/der Untersuchende hält seinen Finger in ca. 90% Reichweite vor der Person. Die Person wird gebeten, mehrmals den Zeigefinger von der eigenen Nase zum Finger der/des Untersuchenden zu bewegen. Die Bewegungen werden in moderater Geschwindigkeit durchgeführt. Die durchschnittliche Ausführung der Bewegungen wird entsprechend der Ausprägung des kinetischen Tremors bewertet.   1. **Kein Tremor** 2. **Tremor mit einer Amplitude von < 2cm** 3. **Tremor mit einer Amplitude von < 5cm** 4. **Tremor mit einer Amplitude von > 5cm** 5. **Durchführen von 5 Zeigebewegungen nicht möglich** | | |
| --- | --- | --- | --- | --- | --- |
| **Punkte** | **R**echts | **L**inks | **Punkte** | **R**echts | **L**inks |
| Punkte Mittelwert beider Seiten  (R+L)/2 | |  | Punkte Mittelwert beider Seiten  (R+L)/2 | |  |
| **7) Schnelle alternierende Handbewegungen**  **Für jede Seite einzeln werten**  Die Person sitzt bequem, eventuell Füße und Rumpf unterstützen. Die Person wird gebeten, 10 Wiederholungen von alternierenden Pro- und Supinationen der Hand auf dem Oberschenkel, so schnell und so genau wie möglich durchzuführen. Die Bewegung wird mit einer Geschwindigkeit von ca. 10 Wiederholungen/7 Sek. vorgezeigt. Die exakten Zeiten für die Bewegungsausführung der Person müssen gemessen werden.   1. **Normal, keine Unregelmäßigkeiten (durchgeführt in  < 10 Sek.)** 2. **Leicht unregelmäßig (durchgeführt in < 10 Sek.)** 3. **Deutlich unregelmäßig, einzelne Bewegungen schwer zu unterscheiden oder unterbrochen (durchgeführt in  < 10 Sek.)** 4. **Sehr unregelmäßig, einzelne Bewegungen schwer zu unterscheiden oder unterbrochen (durchgeführt in  > 10 Sek.)** 5. **Durchführen von 10 Wiederholungen nicht möglich** | | | **8) Knie-Hacke Versuch**  **Für jede Seite einzeln werten**  Die Person liegt auf einer Untersuchungsliege, ohne auf die Beine zu sehen. Sie wird gebeten, ein Bein anzuheben, mit der Ferse das Knie der Gegenseite zu berühren und entlang des Schienbeins zum Knöchel zu gleiten und anschließend das Bein zurück auf die Liege zu legen. Diese Aufgabe wird drei Mal wiederholt. Jede Gleitbewegung sollte innerhalb 1 Sek. durchgeführt werden. Wird die Gleitbewegung bei allen 3 Wiederholungen ohne Kontakt der Ferse zum Schienbein durchgeführt, so wird dies mit 4 bewertet.   1. **Normal** 2. **Leicht auffällig, Kontakt zum Schienbein bleibt erhalten** 3. **Deutlich auffällig, Kontakt zum Schienbein geht bis zu drei Mal über alle 3 Wiederholungen verloren** 4. **Stark auffällig, Kontakt zum Schienbein geht vier Mal oder öfter über alle 3 Wiederholungen verloren** 5. **Durchführen der Aufgabe nicht möglich** | | |
| **Punkte** | **R**echts | **L**inks | **Punkte** | **R**echts | **L**inks |
| Punkte Mittelwert beider Seiten  (R+L)/2 | |  | Punkte Mittelwert beider Seiten  (R+L)/2 | |  |

SARA-Score berechnet sich aus der Summe aller grau hinterlegten Felder (Spanne 0-40; siehe Schmitz-Hübsch et al. 2006.)
kulturelle Anpassung und Übersetzung ins Deutsche: J. Silberbauer, S. Schidl, G. Diermayr, T. Schmitz-Hübsch, A. Greisberger
DOI: 10.1007/s10354-023-01014-8
